# Quantifying the removal of red blood cells in *Macaca mulatta* during a *Plasmodium coatneyi* infection

**DOI:** 10.1186/s12936-016-1465-5

**Published:** 2016-08-12

**Authors:** Luis L. Fonseca, Harnel S. Alezi, Alberto Moreno, John W. Barnwell, Mary R. Galinski, Eberhard O. Voit

**Affiliations:** 1 The Wallace H. Coulter Department of Biomedical Engineering, Georgia Institute of Technology and Emory University, Atlanta, GA USA; 2 Division of Infectious Diseases, Department of Medicine, Emory University, Atlanta, GA USA; 3 Malaria Branch, Division of Parasitic Diseases and Malaria, Centers for Disease Control and Prevention, Atlanta, GA USA; 4 Malaria Host–Pathogen Interaction Center, Emory Vaccine Center, Yerkes National Primate Research Center, Emory University, Atlanta, GA USA

**Keywords:** *Macaca mulatta*, Malarial anaemia, Mathematical model, *Plasmodium coatneyi*, Red blood cell removal

## Abstract

**Background:**

Malaria is the most deadly parasitic disease in humans globally, and the long-time coexistence with malaria has left indelible marks in the human genome that are the causes of a variety of genetic disorders. Although anaemia is a common clinical complication of malaria, the root causes and mechanisms involved in the pathogenesis of malarial anaemia are unclear and difficult to study in humans. Non-human primate (NHP) model systems enable the mechanistic study and quantification of underlying causative factors of malarial anaemia, and particularly the onset of severe anaemia.

**Methods:**

Data were obtained in the course of *Plasmodium coatneyi* infections of malaria-naïve and semi-immune rhesus macaques (*Macaca mulatta*), whose red blood cells (RBCs) were labelled in situ with biotin at the time the infections were initiated. The data were used for a survival analysis that permitted, for the first time, an accurate estimation of the lifespan of erythrocytes in macaques. The data furthermore formed the basis for the development and parameterization of a recursive dynamic model of erythrocyte turnover, which was used for the quantification of RBC production and removal in each macaque.

**Results:**

The computational analysis demonstrated that the lifespan of erythrocytes in macaques is 98 ± 21 days. The model also unambiguously showed that death due to senescence and parasitaemia is not sufficient to account for the extent of infection-induced anaemia. Specifically, the model permits, for the first time, the quantification of the different causes of RBC death, namely, normal senescence, age-independent random loss, parasitization, and bystander effects in uninfected cells. Such a dissection of the overall RBC removal process is hardly possible with experimental means alone. In the infected malaria-naïve macaques, death of erythrocytes by normal physiological senescence processes accounts for 20 % and parasitization for only 4 %, whereas bystander effects are associated with an astonishing 76 % of total RBC losses. Model-based comparisons of alternative mechanisms involved in the bystander effect revealed that most of the losses are likely due to a process of removing uninfected RBCs of all age classes and only minimally due to an increased rate of senescence of the uninfected RBCs.

**Conclusions:**

A new malaria blood-stage model was developed for the analysis of data characterizing *P. coatneyi* infections of *M. mulatta*. The model used a discrete and recursive framework with age-structure that allowed the quantification of the most significant pathophysiological processes of RBC removal. The computational results revealed that the malarial anaemia caused by this parasite is mostly due to a loss of uninfected RBCs by an age-independent process. The biological identity and complete mechanism of this process is not fully understood and requires further investigation.

## Background

Malaria, caused by infection with parasites of the *genus Plasmodium,* is responsible for over half a million deaths per year worldwide, with children being the main victims [1]. The immense severity of the disease and the long coexistence of humans and *Plasmodium* parasites have even led to the emergence and perpetuation of possible protective genetic disorders like thalassaemias and sickle-cell disease, as well as haemoglobin C, haemoglobin E, and G6P dehydrogenase and pyruvate kinase deficiencies [2–7]. In primates, *Plasmodium* sporozoites infect, transform and multiply within parenchymal hepatocytes to form merozoites; these developmental life cycle processes are asymptomatic for the host. Once merozoite forms of the parasite are released from the infected hepatocytes, the parasite begins its cyclical blood-stage development. Merozoites invade and multiply within red blood cells (RBCs) every 24, 48 or 72 h, depending on the species, and new merozoite progeny are released to invade other RBCs [8]. During this cyclical process of invasion and destruction of RBCs, the symptoms and clinical complications associated with malaria emerge. The blood stage of an infection is characterized by repeated rounds of RBC invasion which, if not kept under control by host immune responses or anti-malarial treatment, can lead to exponential growth of the parasite, with a concomitant destruction of the parasitized RBCs. This destruction, however, is not the predominant mechanism of RBC removal that leads to anaemia, and indeed seems to be vastly surpassed by the destruction of uninfected RBCs (uRBCs) [9–11].

Infections with *Plasmodium coatneyi,* a simian malaria species that is closely related to *Plasmodium knowlesi* [12, 13], mirror the biology and pathogenesis of *falciparum* malaria, with severe forms of pathology including anaemia. To study mechanisms of the onset and recovery of anaemia, Moreno et al. [14] established procedures to measure the turnover of in vivo biotinylated RBCs in rhesus macaques (*Macaca mulatta*). These macaques had been experimentally infected for the first time (*i.e.,* when malaria naïve) with *P. coatneyi* infected RBCs and, then again, while partially immune, 9 months after curative anti-malarial drug treatment. Five infected and then re-challenged (semi-immune) animals were compared to five control rhesus with biotinylated RBCs, but no malaria infection. Microscopy-based counts of infected RBCs and haemoglobin levels were monitored daily, and the numbers of biotinylated RBCs were assessed using flow cytometry. This work demonstrated that malarial infections result in an accelerated turnover of uninfected RBCs, and this was most pronounced in the malaria naïve animals. The precise mechanisms causing malarial anaemia are unknown, but have been suggested to be due to multiple possible factors leading to a reduction in circulating RBCs including physicochemical membrane changes, reduced erythrocyte deformability, accelerated erythrocyte senescence, and immunological reactions that cause their removal [14, 15].

This paper presents a mathematical model that was developed to study RBC dynamics in circulating blood during malaria infections. The model was parameterized using experimental results from Moreno et al. [14] and implemented as a discrete recursive structure that was previously identified as best suited for this class of problems [16]. The choice of this framework was based on the need to account for the aging of the RBCs rather accurately, which is problematic in delayed differential and integro-differential equations that do not track age directly, but instead approximate the time passed since the cell was generated. Ordinary differential equations with age classes do not model the aging accurately since inspection of age-classes reveals a distribution of ages within each class. Partial differential equation models like the Lotka–McKendrick age-structured population model [17, 18] do accurately model the aging of a population, but are difficult to implement, especially if the model includes variables with and without an age-structure and if the aging process is perturbed by events like a malaria infection. The most effective alternative is a discrete recursive framework with age-classes. In a sense, this structure corresponds to the discretization of a PDE model and hence shares all of its properties, but is more easily implemented and faster to solve, with an accuracy that is readily tuned by stipulating a desired time-step.

The model permits the quantification of the production of newly generated RBCs and of the different processes leading to the removal of RBCs in the absence or presence of a *P. coatneyi* infection in malaria naïve or semi-immune rhesus macaques. Additionally, two alternative mechanisms of uninfected RBC removal, namely accelerated erythrocyte senescence and immunologic removal, are modelled and compared on the basis of their respective predictions. The results demonstrate that the destruction of uninfected RBCs was the dominant process underlying malarial anaemia in the *P. coatneyi* infections reported by Moreno et al. [14], and that the direct destruction of infected RBCs by the parasite accounted for only about 4 % of the total RBC loss. Beyond this specific result, the model can be employed as a tool for predicting and exploring disease severity and evaluating host-directed interventions. This capability includes the study of other species of *Plasmodium* that cause malaria in primates, each with their unique blood-stage biology and pathogenic characteristics [8].

### Red blood cell removal processes

In healthy humans and non-human primates (NHPs), RBCs are produced by the erythropoietic system in the bone marrow, which is under the control of several cytokines including, in particular, erythropoietin (for a review see [19]). Removal of RBCs is a task for the phagocytic arm of the immune system in response to injury, senescence, or other processes. Injured RBCs are usually removed in the spleen, as they fail to deform and can no longer pass through the microcirculation of the red pulp [20].

Senescence-driven removal of RBCs typically occurs due to oxidative stress to haemoglobin, which results from the continuous cycling between normoxia and hypoxia. This cycling between oxidative states eventually triggers the formation of methemoglobin which ultimately denatures into haemichromes [21]. These haemichromes are able to bind to the cytosolic side of AE1 protein (Band 3) and thus to displace ankyrin, which weakens the AE1 connection to the cytoskeleton and eventually results in clustering of AE1 [22, 23]. On the external side, AE1 protein, which is usually found in dimers, has a low affinity for binding naturally occurring IgG antibodies (NAbs). However, once bound to haemichromes on the inside, AE1 dimers are able to aggregate and bind to the NAbs with enhanced affinity [24, 25]. NAbs alone are not efficient at promoting RBC clearance, but they are able to activate the classical pathway of the complement system and thereby induce erythrophagocytosis [26, 27]. It has also been proposed that AE1 recognition by NAbs may depend on the proteolytic degradation of AE1 [28].

## Methods

### Determination of the hazard function of RBCs in *Macaca mulatta*

**Fig. 1:**
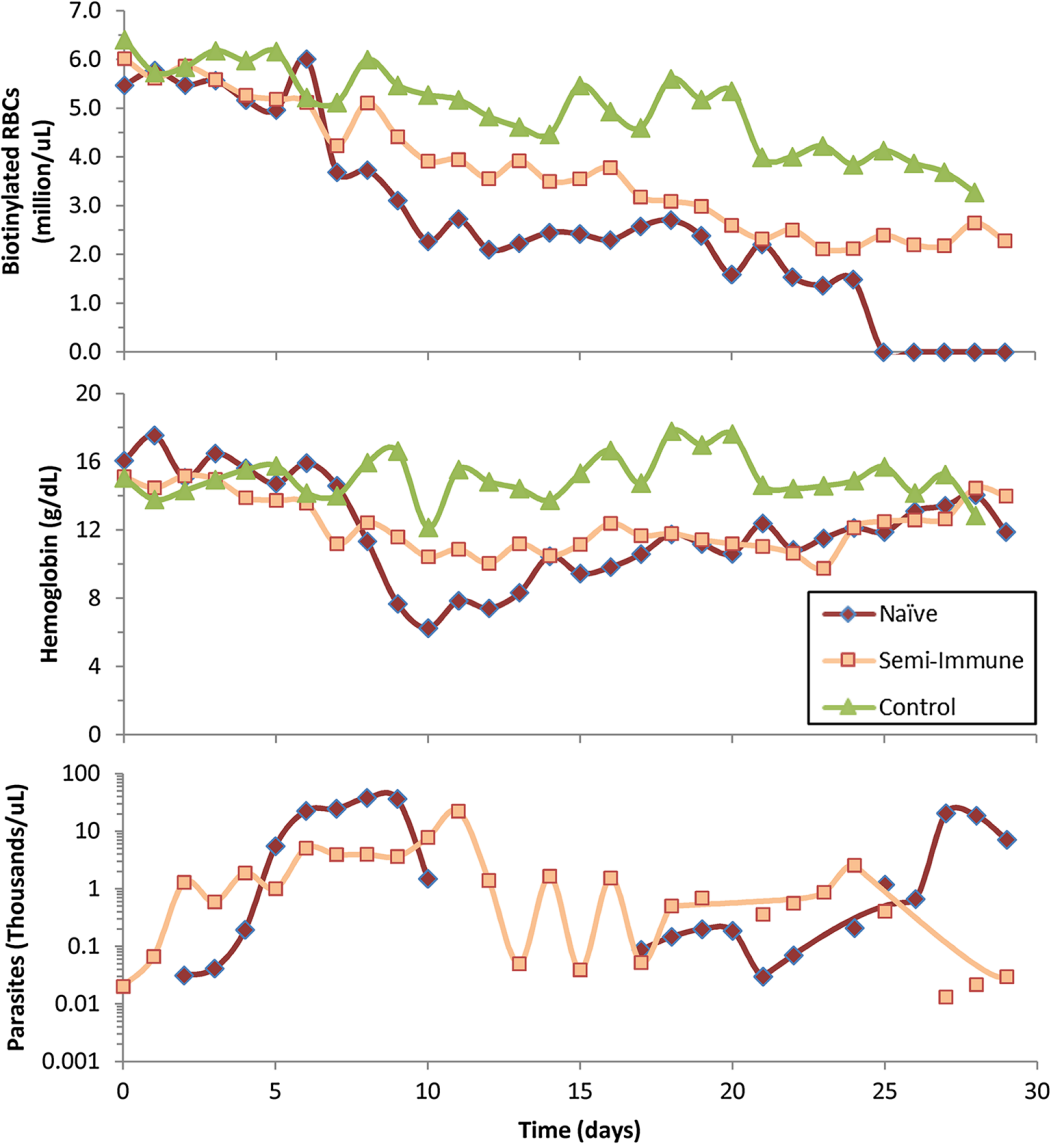
Experimental results redrawn from Moreno et al. [14]. These results were used for the quantification of the RBC production and removal processes. *Data points* represent the means of five rhesus macaques. The parasite levels in the naïve infected group (*bottom panel*) become undetectable after sub-curative treatment on day 10

The time series of biotinylated RBC survival [14] were used to fit a suitable hazard function (Fig. 1a). Different candidate functions were tested, including the typical polynomial and exponential functions (corresponding to the Gompertz survival law [29]). The best fits were obtained with a power-law function, which correspond to a Weibull survival process [30]. As suggested elsewhere [31], an age-independent “Makeham” term was added to account for the age-independent loss already included in the model (see next section and Refs. [32–34]). This term, *c*, was estimated to be 4.36 × 10^−5^.

The resulting hazard function

1 $$h\left( x \right) = c + a \cdot x^{b}$$

is also known as mortality rate, failure rate, or mortality force. It represents the rate of failure (cell death) as a function of age, where *x* is the age of the RBC in hours. In this case, the parameter values were determined by least-squares optimization (Matlab: *fminsearch*) as *a* = 8.488 × 10^−45^ and *b* = 12.25.

The survival curve, which represents the percentage of all cells still alive at a given age, can be readily calculated from the hazard function as [35].

2 $$S\left( x \right) = e^{{ - \int_{0}^{x} {h\left( x \right)dx} }}$$

The probability density function is given by $f = h\, \cdot S$, and it was verified that the corresponding cumulative indeed converges to 1: $\int_{0}^{\infty } {f = 1}$. The predicted survival time course (*Sc*(*t*)) can then be estimated at time *t* as the number of living cells older than *t*:

3 $$Sc\left( t \right) = Prod \cdot \int_{t}^{ + \infty } {S\left( a \right)da} .$$

Here, *Prod* is the hourly production of RBCs. Its value was estimated by least-squares optimization (Matlab: *fminsearch*) of *Sc(t)* against experimental data that were obtained by tracking the surviving biotinylated RBCs in a cohort of five healthy uninfected macaques for over 120 days [14].

### Host-pathogen interaction model in *Macaca mulatta*

A host-pathogen interaction model was developed using a discrete recursive framework with age-classes, as this structure allows effective modelling of the exact amount of time a cell survives [16]. Uninfected RBCs exist in two states. Both populations, unlabelled (*RBC*) and biotinylated (*bRBC*) RBCs, are represented with 3840 age-classes (allowing a maximum lifespan of 160 days or 3840 h, which is intentionally chosen longer than a priori expected to ensure total removal). In the absence of any removal processes, this set-up would generate a rectangular age-distribution with the same number of cells in each class.

The total amount of haemoglobin (*H*) is proportional to the total amount of RBCs (Eq. 4) and dependent on the ratio of haemoglobin to RBC (R_H/RBC_), which is specific to each macaque:

4 $$H = R_{H/RBC} \cdot \left( {bRBC + RBC} \right)$$

The malarial merozoites (*M*) are able to infect RBCs, independently of their biotinylation status, and thus produce infected RBCs (*iRBC*). These, in turn, live for another 48 h, thus occupying 48 age classes, at the end of which they burst and release 20 new merozoites [8, 36]. Exposure of the immune system to infected RBCs leads to the up-regulation of the immune response against infected RBCs (*I*), which in turn triggers the removal of infected RBCs by the spleen.

At the beginning of each simulation, all RBCs are biotinylated, while subsequent erythropoiesis produces exclusively non-biotinylated RBCs. Independent of their biotinylation status, all RBCs are equally subject to the different removal processes, as discussed before. According to the literature [32–34], age-independent (random) death accounts for the normal removal of 10 % of all produced RBCs. This process was included in the hazard function as the constant term *c*. Senescent death was modelled with the power-law term of the hazard function shown in Eq. (1), and removal of uninfected RBCs was modelled as an age-independent process and quantified by least-squares optimization using the experimental data for each of the infected macaques.

### Model formulation

The model has five dependent variables (*RBC*, *bRBC*, *iRBC*, *M*, *I*), of which *RBC* and *bRBC* have 3840 age classes each and *iRBC* has 48 age classes. Additionally, an auxiliary dependent variable, *rRBC*, was used to model the pool of cells lost due to the age-independent (random) process. The variables *M*, *I* and *rRBC* do not have an age-structure.

The pools of *RBC*, *bRBC* and *rRBC* are modelled recursively as follows:

5 $$\begin{aligned} bRBC_{i,t + 1} = bRBC_{{i - r_{SI} ,t}} \cdot \left( {1 - r_{SD} } \right) \cdot Sf_{{i - r_{SI} }} + bRBC_{{i - 1 - r_{SI} ,t}} \cdot r_{SD} \cdot Sf_{{i - 1 - r_{SI} }} , \hfill \\ \left\{ {\begin{array}{*{20}c} {i \in \left\{ {r_{SI} + 1,r_{SI} + 2, \ldots ,3839} \right\}\quad for \,\, r_{SD} = 0 } \\ {i \in \left\{ {r_{SI} + 2,r_{SI} + 3, \ldots ,3839} \right\} \quad for \,\, r_{SD} > 0} \\ \end{array} } \right.\,\,\,\,\,\,\,\,\,\,\,\,\,\,\,\,\,\,\,\,\,\,\,\,\,\,\,\,\,\,\,\,\,\,\,\,\,\,\,\,\,\,\,\,\,\,\, \hfill \\ \end{aligned}$$

6 $$bRBC_{{r_{SI} + 1,t + 1}} = bRBC_{1,t} \cdot \left( {1 - r_{SD} } \right) \cdot Sf_{1} \quad for \,\,r_{SD} > 0$$

7 $$bRBC_{ 1,t + 1} = 0$$

8 $$bRBC_{i,t + 1} = 0, \quad i \in \left\{ {2,3, \ldots ,r_{SI} } \right\}$$

9 $$Sf_{i,t} = \left( {1 - \frac{{RD_{t} }}{TRBC} - hf_{i} - \frac{M}{TiRBC} \cdot IA_{i} - \frac{{uR_{t} }}{TRBC}} \right)$$

10 $$r_{SI,t} = int\left( {r_{S,t} } \right) \wedge r_{SD,t} = r_{S,t} - r_{SI,t} ,\quad r_{S,t} \ge 1$$

11 $$hf_{i} = 8.488 \times 10^{ - 45} \cdot i^{12.25} , \quad i \in \left\{ {1,2, \ldots ,3840} \right\}$$

12 $$\begin{aligned} RBC_{i,t + 1} = RBC_{{i - r_{SI} ,t}} \cdot \left( {1 - r_{SD} } \right) \cdot Sf_{{i - r_{SI} }} + RBC_{{i - 1 - r_{SI} ,t}} \cdot r_{SD} \cdot Sf_{{i - 1 - r_{SI} }} , \hfill \\ \left\{ {\begin{array}{*{20}c} {i \in \left\{ {r_{SI} + 1,r_{SI} + 2, \ldots ,3839} \right\} \quad for \,\, r_{SD} = 0 } \\ {i \in \left\{ {r_{SI} + 2,r_{SI} + 3, \ldots ,3839} \right\} \quad for \,\, r_{SD} > 0} \\ \end{array} } \right. \hfill \\ \end{aligned}$$

13 $$RBC_{{r_{SI} + 1,t + 1}} = RBC_{1,t} \cdot \left( {1 - r_{SD} } \right) \cdot Sf_{1} \quad for\quad r_{SD} > 0$$

14 $$RBC_{1,t + 1} = P_{Ery,t}$$

15 $$RBC_{i,t + 1} = 0,\quad i \in \left\{ {2,3, \ldots ,r_{SI} } \right\}$$

16 $$rRBC_{t + 1} = rRBC_{t} + 0.1 \cdot P_{Ery,t} - \frac{{rRBC_{t} }}{800}$$

17 $$RD_{t} = \frac{{rRBC_{t} }}{800}$$

18 $$uR_{t} = interp1\left( {t,\left[ {1 \ldots 35} \right],DuR_{f} } \right), \quad f \in \left\{ {1,2, \ldots ,35} \right\}$$

19 $$P_{Ery,t} = Prod \cdot interp1\left( {t,\left[ {1 \ldots 35} \right],DP_{f} } \right),\quad f \in \left\{ {1,2, \ldots ,35} \right\}$$

20 $$r_{S,t} = 1 + \frac{{p_{1} }}{{1 + p_{2}^{{p_{3 - t} }} }} \cdot \left( {1 - \frac{1}{{1 + p_{4}^{{p_{5 - t} }} }}} \right), \quad p_{1} , \ldots ,p_{5} \ge 0$$

21 $$TRBC_{t} = \sum\limits_{i = 1}^{3840} {\left( {bRBC_{i,t} + RBC_{i,t} } \right)}$$

22 $$TiRBC_{t} = \sum\limits_{i = 1}^{1200} {\left( {bRBC_{i,t} + RBC_{i,t} } \right)}$$

23 $$IA_{i} = \left\{ {\begin{array}{*{20}c} {1\quad for \,\, i \le 1200} \\ {0\quad for \,\, i > 1200} \\ \end{array} } \right.,\quad i \in \left\{ {1,2, \ldots ,3840} \right\}$$

In this formulation, *r*_*S*_ is the rate of senescence, which has a default value of 1 for the normal rate of senescence where phenotypical age equals chronological age, and a larger value when senescence occurs at a faster rate than the chronological aging of the RBCs. *Sf*_*i,t*_ is the surviving fraction of RBCs in age-class *i* at time-point *t* that will reach the next time point (*t* + 1). The hazard function *hf*_*i*_ was estimated in Eq. (1); it determines the relative number of cells lost from age-class *i*, due to age. *RD*_*t*_ is the total number of cells lost due to random death at time *t* and approximated in Eq. (1) as the age-independent term *c*. *P*_*Ery,t*_ is the rate of erythropoietic production of RBCs at time *t*; it is calculated in Eq. (19) as the product of the specific RBC production rate (*Prod*; which is a property of the healthy state of each macaque) and the daily relative change in erythropoietic production (*DP*_*f*_). *uR*_*t*_ is the number of RBCs lost during an infection from the peripheral blood at time *t* by processes other than parasitization and is calculated by interpolation of the daily number of uninfected RBCs (*DuR*_*f*_) to be removed by these processes. *TRBC*_*t*_ is the total number of healthy RBCs in circulation at time *t*, while *TiRBC*_*t*_ is the total number of susceptible RBCs (younger than 50 days) at time *t*. *IA*_*t*_ is a binary vector of “infectability” for a given age class of RBCs. Equations (5) and (12) allow RBCs in both pools (*bRBC* and *RBC*) to age at a rate faster than their chronological age would normally dictate. This speed-up is achieved by moving cells by *r*_*S*_ age-classes if *r*_*S*_ is integer; if it is not an integer, then the cells in one age class are proportionally moved to two age classes *r*_*SI*_ and *r*_*SI*_ + 1, where the ratio of cells moved to each of these age classes is calculated based on the decimal part of *r*_*S*_, *r*_*SD*_. The closer *r*_*SD*_ is to 1, the larger proportion of cells that are moved by *r*_*SI*_ + 1 age classes. Since RBCs of any age may die at any time point, *Sf*_*i,t*_ (Eq. 9) allows the removal of cells by four different processes: random loss [age-independent, Eqs. (16, 17)]; age-dependent, according to the hazard function, Eq. (11); removal of uninfected RBCs [infection dependent, Eq. (18)] and by parasitization, (Eqs. 22–26). Random loss is modelled in a way similar to the proposal by Löffler’s group [33]. Namely, it is assumed that 10 % of all cells produced will eventually be lost by this random process. Unlike Löffler’s model, it is assumed here that the proportion of cells lost randomly is fixed at 10 % rather than being a function of the number of cells being produced. This change was made because a variable ratio caused the model to become unstable under some conditions. The 10 % of cells produced accumulate in an auxiliary variable (*rRBC*) from where they are removed (Eq. 16) with a first-order rate of (800 h)^−1^. This rate was adjusted from the rate of (1020.4 h)^−1^ for humans [33], based on the fact that the lifespan of rhesus macaque RBCs is approximately one fifth shorter than in humans (100 and 120 days, respectively). The number of cells removed from this auxiliary variable (rRBC) is recorded with Eq. (17), which is then used to estimate the number of cells that are uniformly removed from all age classes of RBCs (Eq. 9).

The *iRBC*, *M* and *I* pools are recursively modelled as:

24 $$iRBC_{i,t + 1} = iRBC_{i - 1,t} \cdot \left( {1 - \frac{{k \cdot I_{t} }}{TiC}} \right),\quad i \in \left\{ {1,2, \ldots ,47} \right\}$$

25 $$iRBC_{ 1,t + 1} = M_{t}$$

26 $$M_{t + 1} = 20 \cdot iRBC_{48,t} \cdot \left( {1 - \frac{{k \cdot I_{t} }}{TiC}} \right)$$

27 $$I_{t + 1} = I_{t} \cdot \left( {1 + \frac{{s \cdot TiC_{t} }}{{\varphi + TiC_{t} }}} \right) \wedge I_{t = 0} = 1$$

28 $$TiC_{t} = \sum\limits_{i = 1}^{48} {\left( {iRBC_{i,t} } \right)}$$

Here, *k* is the rate of iRBC clearance by the immune system; *s* is the maximal rate of the action of the immune response; and $\varphi$ is the number of iRBCs that produce half-maximal action of the immune response.

### Quantification of the increased removal or senescence rate of uninfected RBCs

The quantification of the production and removal of uninfected RBCs was performed through non-linear regression analysis using the experimental data from Moreno et al. [14] (Fig. 1).

Control macaques were simulated by considering that the daily rate of uninfected RBC removal (*DuR*_*f*_) is zero and the daily relative erythropoietic production (*DP*_*f*_) is one:

29.1 $$DuR_{f} = 0, \quad f \in \left\{ {1,2, \ldots ,35} \right\}$$

29.2 $$DP_{f} = 1, \quad f \in \left\{ {1,2, \ldots ,35} \right\}$$

29.3 $$r_{S,t} = 1,\quad for \,\,\,\forall t > 0$$

29.4 $$M_{t} = 0,\quad for \,\,\forall t > 0$$

For each control macaque, the specific RBC production rate [*Prod*; Eq. (19)] and haemoglobin-to-RBC ratio (*R*_*H/RBC*_) were obtained by least-squares optimization of the trajectories for the total number of biotinylated RBCs $\left( {TbRBC_{t} = \sum\nolimits_{i = 1}^{3840} {\left( {bRBC_{i,t} } \right)} } \right)$ and the haemoglobin levels $\left( {H_{t} = R_{H/RBC} \cdot TRBC_{t} } \right)$ against the experimentally obtained time-series data for these variables.

The characterization of the infection of rhesus macaques was performed in several steps:

1. The infected macaques were simulated first as control macaques. Then the time of infection (*toi*) and the corresponding initial number of infected cells (*noi*) were determined such that
  30 $$M_{t = toi} = noi$$
  results in a simulated trajectory for the iRBCs that approximates the initial growth of the infected RBCs. The control and elimination of the infection were obtained by optimization of the parameters *k*, *s* and $\varphi$ (Eqs. 24, 27) for the semi-immune, infected macaques. For the naïve macaques, a sub-curative treatment was administered at peak parasitaemic periods post-infection as these macaques were otherwise not able to control their acute parasitaemias and would probably have died. The solutions for the parameters *toi*, *noi*, *k*, *s* and $\varphi$ were not unique and the actual values were therefore not interpreted biologically. They were used exclusively as a means to simulate the macaque-specific parasitaemia levels.
2. Next, the macaque-specific RBC production rate (*Prod*) and haemoglobin-to-RBC ratio (*R*_*H/RBC*_) were obtained by least-squares optimization against the first five time-points available for the biotinylated RBCs and haemoglobin levels of each macaque.
3. The mechanism-dependent parameters were then obtained in a mutually exclusive way.
  3.1. Age-independent mechanism: The experimentally obtained time series data for the biotinylated RBCs were fitted by optimization of daily levels of uninfected RBC removal ($DuR_{f} ,\quad f \in \left\{ {1,2, \ldots ,30} \right\}$) while keeping *p*_1_ = 0 (Eq. 20).
  3.2. Increased senescence mechanism: The rate of senescence [*r*_*S*_: *p*_1_*,…,p*_5_ in Eq. (20)] was fitted against experimentally obtained time-series data for the biotinylated RBCs, while keeping $DuR_{f} = 0\,\,( f \in \left\{ {1,2, \ldots ,35} \right\}$).
4. The levels of erythropoietic up-regulation of RBC production ($DP_{f} , \,\,f \in \left\{ {1,2, \ldots ,30} \right\}$) were optimized by fitting the experimental time-series data for the haemoglobin levels.
5. A re-fitting of *DuR*_*f*_ and *DP*_*f*_ (age-independent model) or of *r*_*S*_ and *DP*_*f*_ (senescence model), using the previous results (2.1 and 3 or 2.2 and 3) as starting points, against the experimental biotinylated RBC numbers and haemoglobin levels was performed to obtain the best overall fit.

## Results

### Modelling the life span of RBCs

**Fig. 2:**
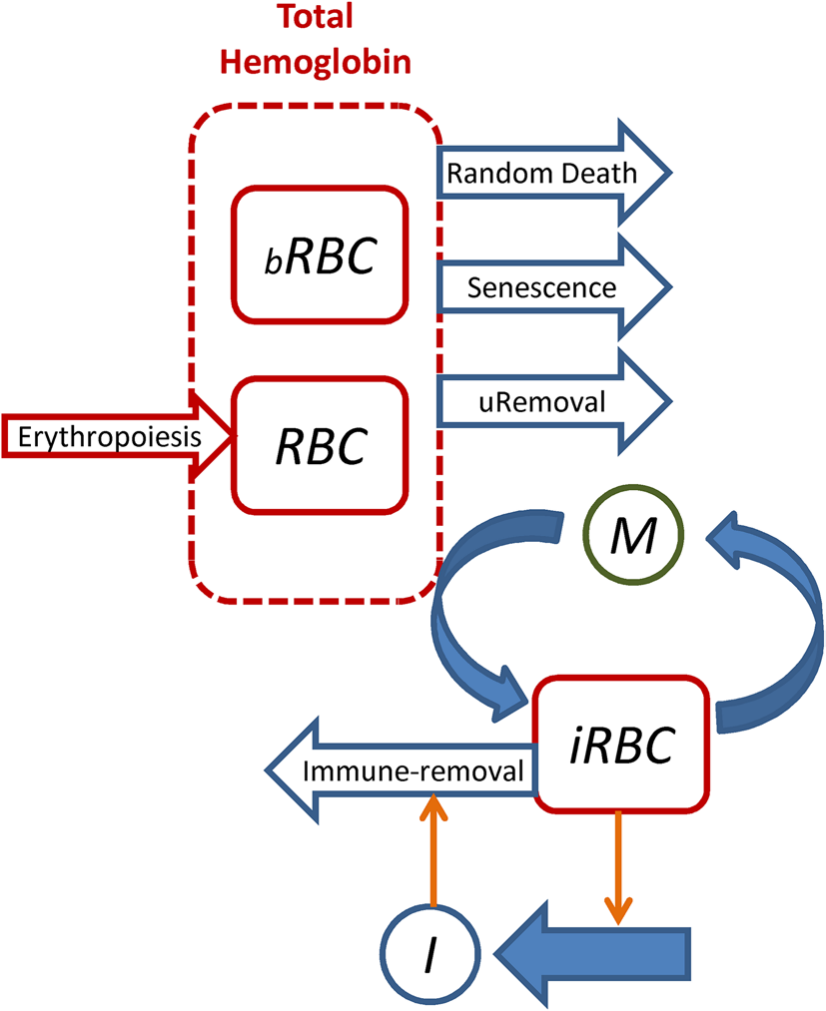
Model scheme of RBC turnover. Two pools of RBCs are modelled, namely *RBC* (unlabelled RBCs) and *bRBC* (biotinylated RBCs), the sum of which equals the total number of RBCs and is proportional to the total amount of haemoglobin present. Production of RBCs by erythropoiesis only increases the unlabelled RBC pool (*RBC*). Both pools of RBCs (*RBC* + *bRBC*) are prone to removal by four processes: age-dependent death (senescence), age-independent (random) death, removal of uninfected RBCs (uRemoval), and removal due to parasitization. Parasitization occurs when a merozoite (*M*) infects a RBC, thus becoming an infected RBC (*iRBC*). Infected RBCs stimulate an immune-response (*I*), which in turn leads to the removal of infected RBCs. At the start of a simulation, all RBCs are biotinylated. All pools of RBCs have age-classes, which are not depicted in the scheme for simplicity. Unlabelled and biotinylated RBCs have 3840 age-classes, whereas infected RBCs have only 48 age-classes

A recursive model of the dynamics of circulating RBCs in rhesus macaques was developed by discretization of both time and age. Here, the terminology of “RBCs” refers to mature red blood cells and does not include reticulocytes. In short, two pools of RBCs are modelled with 3840 age-classes each (Fig. 2). One pool represents unlabelled RBCs (*RBC*), while the second pool contains biotinylated RBCs (*bRBC*). It can be reasonably assumed that the spleen and immune system treat both types in the same manner.

In the beginning of the experiments that are modelled here, all RBCs are labelled (Fig. 1). Therefore, initially only the biotinylated pool contains cells, while production of new RBCs only occurs at the first age-class of the unlabelled pool (Erythropoiesis, Fig. 2). Both pools are assumed to be subject to the same hazard function, which is represented as Eq. (11) in the “Methods” section and with parameters estimated in the “Survival analysis of RBCs in *M. mulatta*” section. This hazard function (Eq. 1) accounts for the two normal processes of RBC removal present in a healthy host, namely age-dependent death due to senescence, and age-independent death. The latter type of destruction will be referred to as *random*, which follows the terminology of the field [32, 33, 37], even though it is unclear whether the process is truly stochastic or whether it is governed by deterministic processes that are not well understood. This age-independent random death is modelled under the assumption that 10 % of all RBCs produced will eventually die by this process (Eqs. 16, 17). The initial age distribution of bRBCs is calculated by allowing the model to run to steady state, and the age distribution of the variable *RBC* (unlabelled RBCs) is recorded in this state.

To model the blood stage of a malarial infection, a pool of merozoites (*M*) is included with a single 1-h age class since, once released from infected RBCs (*iRBCs*), merozoites target and infect available uninfected RBCs within a matter of minutes. Also taken into account is a pool of infected RBC (*iRBC*) with 48 age classes, corresponding to the 2 days of the intra-erythrocytic lifespan of *P. coatneyi*. To mimic the body’s response to the infection, an immune response (*I*) is introduced as a black box. It acts such that exposure to parasite antigen represented by the *iRBC* leads to an increase in the immune response (*I*), which in turn kills iRBCs in a process that is linearly dependent on the number of iRBCs and on the immune response (Immune-removal, Fig. 2; Eq. 24). Even with this small number of processes representing the immune response, the model does not have a unique solution for each of the macaques in terms of immune-related parameters. For this reason, no other processes, such as immune exhaustion, were included and the numerical values of the parameters will not be discussed, as this immune module is thought to be a utilitarian module without a specific foundation for biological interpretability.

### Survival analysis of RBCs in *Macaca mulatta*

**Fig. 3:**
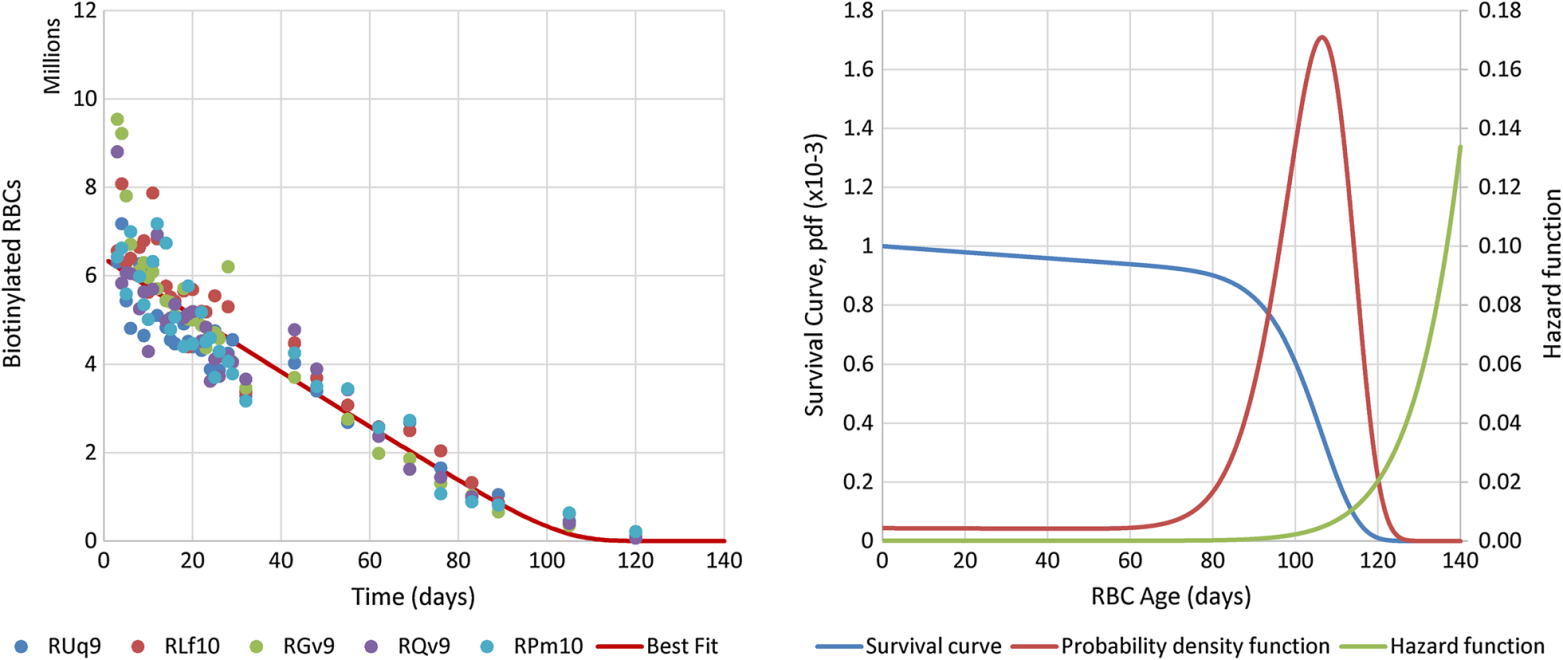
Determination of the hazard function for RBCs in healthy rhesus macaques. The *left panel* shows the experimentally determined time courses of biotinylated RBC survival for five healthy rhesus macaques (*dots*). The *red line* depicts the best fit time course for the biotinylated RBCs survival, which was obtained from the hazard function (*green*) in the panel on the *right*. This* panel* also exhibits the corresponding survival curve (*blue*) and the associated probability density function (*red*). From the probability density function, an average RBC lifespan of 98 ± 21 days was calculated

Survival analysis was performed to characterize the removal of RBCs under normal, healthy conditions. To this end, a hazard function was calculated for the RBC survival data obtained in five control macaques studied by Moreno et al. [14] (Fig. 3). Several functional forms were tested, including polynomial and exponential functions, but best fits were obtained for a power-law representation, which corresponds to the so-called Weibull survival law [30]. Parameterization of the hazard function (Fig. 3b) was done by least-square optimization against the RBC survival data (Fig. 3a). The best hazard function and associated survival and probability density functions are shown in Fig. 3b. Analysis of the probability density function (Fig. 3b) yields an average RBC lifespan of 98 ± 21 days.

### Quantification of the enhanced removal of uninfected RBCs

The model distinguishes and allows the quantification of RBC loss due to four distinct processes (Eq. 9): age-dependent elimination due to senescence, age-independent random removal, parasitization, and removal of uninfected RBCs by other means. Age-independent removal of RBCs is assumed to be constant, because it is known that 10 % of all newly formed RBCs will be removed at some unpredictable time point before the natural life span is reached (see [16, 32, 33]). Senescent removal is modelled in accordance with the hazard function, which was determined from healthy rhesus macaques (Fig. 3b). Loss of RBCs due to parasitization refers to RBCs that are infected by merozoites and subsequently destroyed upon maturation of the new intracellular parasite progeny. The designation of *removal of uninfected RBCs by other means* excludes all of the other removal processes and is required in order to match the experimental data. For the non-infected control macaques, the model takes into account only the first two processes: age-independent removal and senescence of RBCs.

**Fig. 4:**
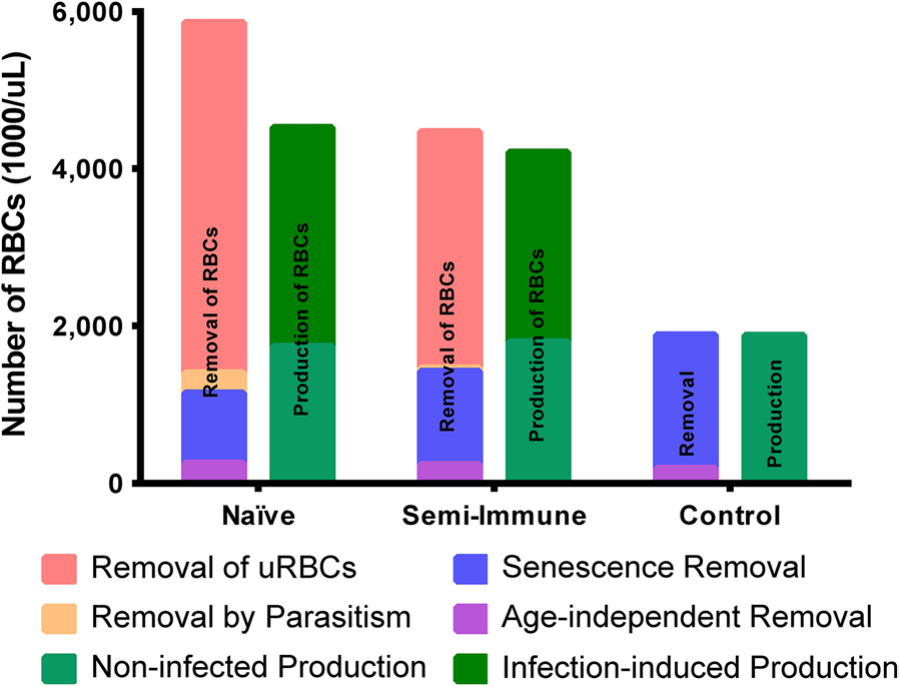
Comparison of the average levels of RBC production and removal calculated for malaria naïve infected, semi-immune infected, and control rhesus macaques. The *plot* shows the total amount of production and removal by the different processes affecting RBCs during the 30-day period when the macaques were followed. In the infected macaques, the production was separated into two components: the amount of erythropoietic output the macaques would have had if they had not been infected (Non-infected Production) and the production of RBCs induced by the infection. Four different removal processes were quantified, two of which are normal physiological processes: Senescence Removal and Age-independent Removal. Here, age-independent removal accounts for the normal lysis of RBCs, which occurs in circulation due to the physical stresses imposed on the RBCs, but which are not dependent on age. Senescence removal accounts for all normal processes by which RBCs are taken from circulation due to age. The two removal processes that are due to the infection with *Plasmodium* are direct removal due to parasitization and removal of uninfected RBCs. The former includes only cells that are infected by the parasite, whereas the latter accounts for RBCs that are removed during an infection but were not infected by a merozoite

The model was fitted to the experimental longitudinal data—that is, the numbers of biotinylated RBCs, haemoglobin levels, and parasitaemias—from each of the macaques, by optimization of the daily RBC production and removal rates. The total numbers of cells produced or lost by the different processes during the experimental 30-day period were calculated for each macaque, and the averages for each group of macaques are shown in Fig. 4.

Control non-infected macaques lost RBCs only due to age-independent (random) and age-dependent (senescence) processes (Fig. 4); in these macaques, the production of RBCs matches exactly the natural loss of cells. The infected malaria-naïve macaques (i.e. not infected previously), by contrast, lost more cells than they produced. Most of their RBC loss is accounted for by the elimination of uninfected RBCs (76 % of total), whereas only a small portion was actually parasitized (4 % of total, or 5 % of the infection-induced loss). Relative to control macaques, infected naïve and semi-immune macaques lose fewer RBCs due to senescence (Fig. 4), simply because so many cells are being removed by other processes that considerably fewer RBCs reach old age. Comparatively however, more cells in both naïve and semi-immune infected macaques are lost through age-independent processes (Fig. 4). The reason is that more cells are being produced in these macaques in response to the infections and 10 % of these cells are removed by age-independent process.

The infected semi-immune macaques had milder parasitaemias (Fig. 1) and, therefore, lost fewer cells to parasitization (1 % of total, *p* < 0.001) than the malaria-naïve infected macaques (Fig. 4). Similarly, they also experienced less cell loss due to the removal of uninfected RBCs, although the difference is not significant (*p* < 0.11). The infected macaques, regardless of whether they were infected when naïve or semi-immune, also exhibited a higher erythropoietic output than the control macaques (*p* < 0.007 and *p* < 0.001, respectively) but the difference between the two infected groups is not significant (*p* < 0.42). The term “Non-infected Production” in Fig. 4 denotes the number of RBCs produced by the infected macaques if they had not been infected; thus, production above this level is required to compensate for the infection-induced destruction. The “non-infected production” of RBCs is not significantly different among the three groups evaluated here (malaria naïve, semi-immune, or control). Strikingly, the infection caused an increase in total RBC production to 290 and 235 % in the naïve and semi-immune infected macaques, respectively, relative to their corresponding production in the absence of infection (Fig. 4).

### Model validation

**Fig. 5:**
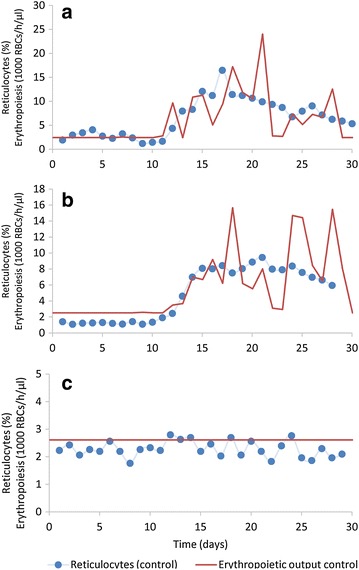
Comparison of time-courses of the inferred erythropoietic output and experimental reticulocytes. The *red lines* represent the average erythropoietic outputs determined for each of the macaques, while the *blue dots* indicate the average of measured reticulocyte levels for each designated experimental group: **a** malaria naïve infected, **b** semi-immune infected, and **c** control macaques

All experiments described here were used to quantify the RBC production and elimination processes; no other comparable experiments are currently available for validation. However, within each of the infection experiments (infection of the macaques when naïve to malaria, and then again later when semi-immune), Moreno et al. [14] also determined the daily percentages of reticulocytes observed in peripheral blood smears. Analyzing these data lends support to the model, because the time-points showing increases in erythropoietic output, as predicted by the model, coincide with the experimentally determined rises in reticulocyte release from the bone marrow into circulation (Fig. 5).

### The process of removal of uninfected RBCs

**Fig. 6:**
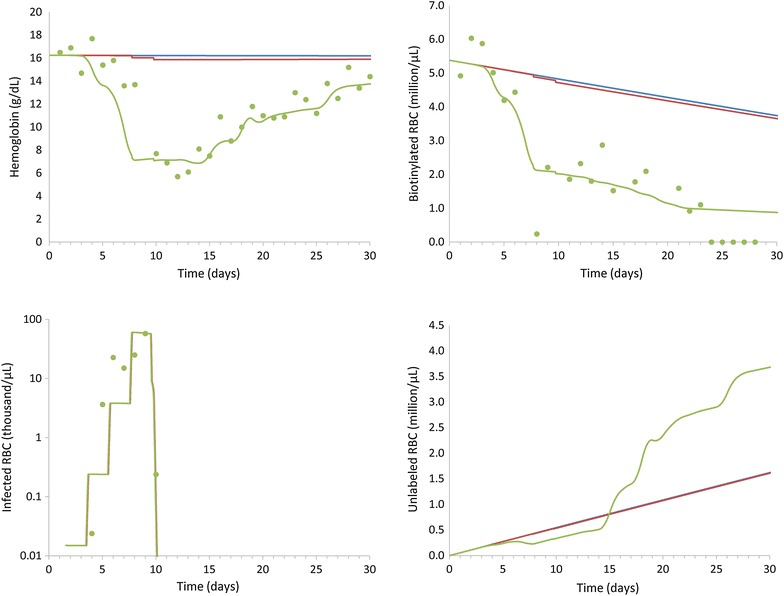
Model simulations comparing cases of no infection, infection alone, and infection with removal of uninfected RBCs and up-regulation of erythropoiesis. Simulation of a healthy, non-infected macaque is shown as a *blue line*. In a healthy macaque, biotinylated RBCs are only slowly lost due to senescence and age-independent processes, while erythropoiesis compensates the loss by producing unlabelled RBCs in such a way that no significant change in haemoglobin is seen. Simulation of an infected macaque, without taking into account loss of uninfected RBCs, is shown as a *red line*. Relative to the healthy macaque, only a small number of biotinylated and unlabelled RBCs are removed by parasitization (mainly at around days 8–10) and infected RBCs are produced. These results do not match the experimental results (*circles*) for haemoglobin and biotinylated RBCs. However, if an infected macaque is simulated taking into account loss of uninfected RBCs and up-regulation of erythropoiesis *green line*, then these processes can be estimated such that the model captures the behavior of the experimental data (*circles*) obtained for that particular animal

If the destruction of RBCs is modelled entirely as due to old age (according to the hazard function), age-independent (random) removal, and parasitization by merozoites, then the levels of anaemia predicted by the model are always much smaller than the values measured by Moreno et al. [14] (Fig. 6). To assess the malarial anaemia properly, two different mechanisms were explored with the same model. To investigate the age-independent mechanism it was assumed that uninfected RBCs are removed during the malarial infection with the same rate, independent of labelling or age; in other words, all uninfected RBCs are equally likely to be removed (Eq. 18). By contrast, the increased senescence mechanism was modelled such that uninfected RBCs age at a faster rate than normal. This increase in rate is modelled in the following manner: Under normal conditions, all RBCs move from one age-class to the next in 1-h time intervals. During a malarial infection, the uninfected RBCs are allowed to age at a faster rate (Eq. 20), by skipping age classes, which subjects them to a higher rate of removal (according to the hazard function) earlier, which consequently shortens their lifespan. Expressed differently, when the rate of senescence is increased, the phenotypic age becomes greater than the chronological age.

**Fig. 7:**
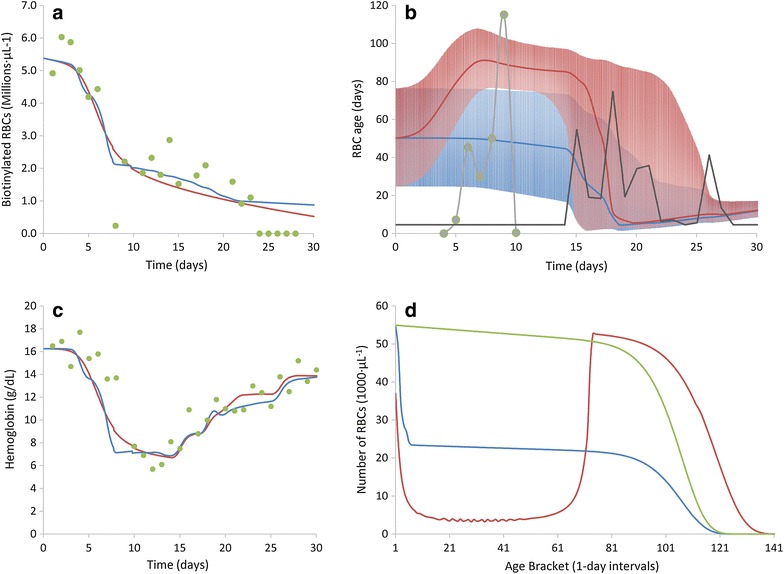
Comparison of fits and predictions of the two mechanistic hypotheses regarding the removal of uninfected RBCs, namely the age-independent model and the increased senescence model. **a** The time course of biotinylated RBCs predicted by each model (*blue line* age-independent model and *red line* increased senescence model), superimposed on the experimental results (*circles*) by Moreno et al. [14]. **b** Exhibits the time courses of the median ages of the RBC populations, predicted by each model (*blue lin*e age-independent model and *red line* increased rate of senescence model). The *shaded areas* highlight the 25th and 75th percentiles of the age distributions of each RBC population. The *light gray line* highlights the time course of parasitaemia, and the *dark grey line* exhibits the time course for the erythropoietic output as predicted for the age-independent model. **c** The haemoglobin time courses predicted by each model (*blue line* age-independent model and *red line* increased senescence model), along with experimental results (*circles*) by Moreno et al. [14]. **d** Exhibits the age-distributions of RBC populations for the age-independent model (*blue line*) and for the increased senescence model (*red line*) at day 9, which corresponds to the highest parasitaemia level (see *panel* **b**). For comparisons, the age-distribution of a healthy RBC population (*green line*) which corresponds to the initial state (day 0) of both models, is also shown

The two hypothesized mechanisms performed equally well with respect to reproducing the experimental data [14] (Fig. 7a, c), as both predict the same rate of RBC removal by a process other than parasitization. The two mechanisms do, however, predict quite different age distributions for the uninfected RBCs during the infection (Fig. 7b). Figure 7d shows the age distributions of RBCs predicted by the two mechanisms for day 9, which coincides with peak parasitaemia, and in comparison with the age distribution of RBCs in the healthy state (day 0) for a representative macaque of the infected malaria-naïve group.

## Discussion

This work introduces a mathematical model that allows for the disentanglement of concurrent processes relating to RBC removal and malarial anaemia, based on published experimental data from a longitudinal malaria infection study with NHPs [14]. The main results present a clearer picture of the life span of RBCs in rhesus macaques and the quantification and age-characterization of the enormous proportion of uninfected RBCs that are eliminated coincident with the rise in parasitaemia and progression of the disease.

Developing appropriate models for such purposes requires a flexible computational framework capable of accounting for large numbers of cells in their proper age classes. In particular, a requirement here was that cells could normally be kept in various age classes for more or less fixed amounts of time, but that they could also skip age classes or remain longer within the same classes in certain situations. One approach of addressing such a situation could be based on delay differential equations (DDEs). However, it was shown elsewhere that DDEs are not flexible enough for modelling malarial anaemia at the cellular level [16]. Delays could also be generated with ordinary differential equations (ODEs) with age-classes, but these do not represent the transitions between age classes well. In contrast to these standard approaches, discrete recursive equations with age classes are not only able to generate hard delays but can also represent the age structure correctly, as can be seen by the fact that all cells in a given age class have the same age [16].

Building upon these advantages, a discrete system was constructed with age classes and a hazard function was added to account for death. This structure allowed an effective representation of the age distribution of RBCs. In the absence of cell death according to the hazard function, the cells would have a rectangular age distribution with constant death rate. Instead, use of a data-driven hazard function allowed cell removal to be modelled in a manner that is very close to reality. The hazard function that fitted the experimental results best and was used here is a power-law function, which captures the fact that cells of all ages can die, but that the number of cells dying at a young age is very small. By the same token, the older a cell becomes the more likely it will die, and the power-law hazard function captures this behaviour well (Fig. 3b).

Two mechanisms were tested to explain the removal of uninfected RBCs during a malarial infection by processes beyond the normal physiological age-dependent and age-independent processes and the direct parasitization of RBCs. These mechanisms underlying the loss of uninfected RBCs, sometimes referred to as the bystander effect, corresponded to two hypotheses: (1) that all cells are equally likely to be removed, independent of age; or (2) that the removal occurs by normal senescence, but that during high parasitaemia levels uninfected RBCs age faster. The age-independent mechanism would seem to be the better choice if bystander RBC removal occurs due to age-independent loss of uninfected RBCs. This removal could potentially result from targeting of the RBCs by the immune system. Specifically, upon rupture of the infected RBCs and release of the new brood of merozoites, the intracellular contents of the RBC and the parasites are released into the circulation and can adhere to uninfected RBCs, thereby potentially making them targets for erythrophagocytosis.

By contrast, an increased senescence mechanism would be more appropriate if the processes leading to the removal of uninfected RBCs were in fact age-related, for example, with an increased rate of senescence of the uninfected RBCs in the face of an infection. For instance, the immune response to the infection could lead to an increased level of oxygen radicals, which in turn could trigger an increased rate of RBC senescence.

The age-independent and the increased senescence mechanisms both fit the experimental data equally well (Fig. 7). However, they very clearly predict a different age distribution of the RBCs, in particular during the peak of parasitaemia (Fig. 7d). While the age-independent mechanism predicts almost no change in the age distribution of RBCs during peak parasitaemia, the increased senescence mechanism predicts a population of RBCs that appears to be much older. It has been reported that uninfected human RBCs co-cultured in vitro with *P. falciparum* infected RBCs have a higher proportion of older cells than same-donor control cultures [38]. Although a similar trend has been observed here (Fig. 7) based on in vivo data, the results [38] suggest a different magnitude of increase in older cells, which in turn implies that increased senescence is neither likely the only—nor a major—process leading to the increased removal of uninfected RBCs.

Thus, the age-independent mechanism appears to be more likely an explanation for quantifying the removal processes. Considering this mechanism, the model allows the differentiation between four distinct processes of RBC removal. Two of these processes are normal physiological processes of RBC loss occurring in healthy macaques, namely, age-dependent and age-independent ‘random’ removal of RBCs. The age-dependent process collectively captures the normal processes of RBC senescence, whereas the age-independent processes encompass all RBC losses that occur under normal physiological conditions but are not related to age. This process was modelled as proposed by Löffler’s group [16, 32, 33], where 10 % of all produced RBCs are destined to be lost by this ‘random’ process.

The remaining two distinct processes of RBC removal are directly and indirectly due to the malarial infection. These are the direct parasitization with the consequential destruction of the infected RBCs and the loss of uninfected RBCs as a bystander effect. RBCs removed by direct parasitization are those RBCs that are invaded by a merozoite for the production of the next generation of merozoites, and destroyed concomitantly with their release into the bloodstream. The estimation of the number of infected cells in the current model is based on the experimental parasitaemia measurements by Moreno et al. [14]. Uninfected RBC removal occurs during the malaria infection for reasons that are not well understood and clearly warrant further investigation, because this process dominates the manifestation of malarial anaemia, as exemplified by the case studied here of *P. coatneyi* infecting *M. mulatta*. In both malaria-naïve and semi-immune infected macaques, the loss of uninfected RBCs emerged as the leading process in RBC removal (76 and 67 % of total removal, respectively), whereas the actual destruction of RBCs by parasitization represented only 4 and 1 % (respectively) of the total removal. The remaining losses were 15 % to senescence, and 4 % to age-independent processes, in infected naïve macaques.

RBC production during the 30-day infection of the malaria-naïve macaques was increased by a factor of almost two, which however did not match the total number of cells lost during this period (Fig. 4). The cause of this discrepancy is reflected in the experimental data, as some of the macaques did not fully recover from their anaemic state within the 30-day experimental interval and after 30 days still exhibited decreased haemoglobin levels (Fig. 1). A much smaller difference between the removal and replenishment of RBCs was also inferred for the semi-immune macaques (Fig. 4), as these macaques were mostly able to recover from their anaemias (Fig. 1). Comparison of the inferred RBC production profiles with the experimentally determined percentage of circulating reticulocytes (Fig. 5) showed good agreement, which attests to the model’s ability to predict correctly when erythropoiesis or release of reticulocytes from the bone marrow was upregulated. Yet, closer analysis reveals that this upregulation does not kick in during the time period when RBCs are being actively removed, but only once parasitaemia decreases due to host immune responses or anti-malarial treatment. This discrepancy suggests that the parasite does not reduce erythropoietic output, but that it rather prevents effective erythropoiesis with the normal release of reticulocytes in response to anaemia; only after the parasite is cleared from circulation is the host able to properly respond to the anaemia. Clinically, the infected macaques (whether naïve or semi-immune) showed evidence of erythroid hyperplasia (expansion of erythroid progenitors) in their bone marrow, coincident with the observed peaks of parasitaemia. However, the erythroid hyperplasia was neither reflected by a high number of reticulocytes in the peripheral blood (experimentally measured in [14]) nor by an increased production of RBCs (inferred in this work), corroborating the hypothesis of parasite-induced ineffective erythropoiesis. Consistent with ineffective erythropoiesis, bone marrow biopsies in naïve macaques showed histological signs of dyserythropoiesis in the form of defective development of erythroid cells [14]. Relevantly, macaques that had been malaria naïve when infected exhibited erythropoietin levels that were elevated six-fold during periods of high parasitaemia, indicating that ineffective erythropoiesis is not mediated by the dysregulation (i.e., lack) of compensatory erythropoietin production [14].

It appears that this article is the first to quantify the percentage of removal of uninfected RBCs accurately in an in vivo primate malaria system. It is moreover likely that the results have implications for understanding anaemia in humans that is caused by *P. falciparum* (and perhaps other species of *Plasmodium*). Two earlier, somewhat indirect studies have attempted to quantify the removal of uninfected and parasitized RBCs in humans infected with *P. falciparum* [10, 11]. Jakeman et al. [10] used a discrete mathematical model to calculate the ratio of uninfected erythrocytes destroyed per infected erythrocyte in twelve neurosyphilis patients who were infected with different strains (McLendon and El Limon) of *P. falciparum* as a treatment (malariotherapy). The results showed that on average 8.52 (1.28–19.28) uninfected RBCs are removed per infected RBC [10]; thus, the loss of uninfected RBCs represents about 90 % of the total removal. Price et al. [11] compared the total loss of RBCs inferred from the decrease in haematocrit, with an estimation of the number of RBCs infected and destroyed by parasites inferred from the parasitaemia level. The authors concluded that parasitization was responsible for 7.9 % (6.2–9.6 %) of the RBCs lost in patients with uncomplicated falciparum malaria [11]. These results are in the same range as our average estimates of 95 % (90–97 %) and 99 % (97–99 %) loss of uninfected RBCs in malaria-naïve compared to semi-immune macaques infected with *P. coatneyi*, relative to the total loss of RBCs induced by the infection.

The physiological basis for the high loss of uninfected RBCs is not known. In this paper, two mechanisms are compared: one where any uninfected bystander RBC is prone to removal and one where the infection causes an increased rate of aging and thus the senescence-based removal of uninfected RBCs. If only the second mechanism is in effect, then the model predicts a rather old population of RBCs during high parasitaemia (Fig. 7c, d). This prediction is at odds with experimental evidence obtained in RBC in vitro cultures infected with *P. falciparum* (D10 strain), which suggested a slight (~10 %) decrease in young uninfected RBCs and a small (~5 %) increase in old uninfected RBCs in comparison with a non-infected control culture of RBCs from the same donor [38]. By direct inference, increased senescence would not seem to be the only—or even the predominant—mechanism responsible for the loss of uninfected RBCs during a malarial infection.

Among the age-independent processes that could be responsible for this removal of uninfected RBCs, it has been suggested that reduced deformability, increased oxidation of membranes, inflammatory insults, or surface deposition of parasite proteins like PfRSP-2 could be involved [39]. Also of interest, data obtained in a model of chronic anaemia in semi-immune BALB/c mice infected with the *P. berghei* ANKA strain showed that this increased rate of removal of uninfected RBCs can be delayed by the depletion of macrophages, thus suggesting an immunopathological process where CD4^+^ T cells may be involved [40].

The model put forth here does not account for the microvascular sequestration of parasitized RBCs during the trophozoite and schizont stages of development, a phenomenon that was not experimentally quantifiable with the data, which targeted specifically the dynamic assessment of peripheral blood and bone marrow samples [14]. The daily monitored, circulating ring-stage infected RBCs can be representative of the parasite burden, but this does not reflect the daily parasite load in the host. Including RBC sequestration in the models would slightly change the rate of death by direct parasitization of RBCs and decrease the rate of removal of uninfected RBCs through the bystander effect. However, the overall results would not change much. As an example, consider the case of malaria-naïve macaques where, during a *P. coatneyi* infection, 5 % of RBCs died by parasitization and 95 % were removed as uninfected RBCs. Even if an equal number of infected RBCs were sequestered in the tissue microvasculature and in the peripheral blood circulation, the levels of death by parasitization would merely rise to 10 % and reduce the removal of uninfected RBCs to 90 %. This scenario does not change the main conclusions of this study, and the removal of uninfected RBCs would still greatly outnumber the cell losses due to parasite invasion of the RBCs.

Another assumption of the model, which could be construed as unlikely to occur in humans and in macaques, is the occurrence of random destruction of RBCs as a normal physiological process of RBC loss. This process was here assumed to have a magnitude of 10 %. If the model had been developed without this process, the parameter *c* in the hazard function (Eq. 1) would be zero, and the parameters *a* and *b* would easily compensate for the absence of *c*. As a consequence, the survival curve shown in Fig. 3b (blue) would not exhibit the slight slope present between the RBC ages of 0 and 60 days, but would be essentially flat in this range. Nevertheless, given that the same number of cells would still have to be removed, as it is evident from the biotinylated RBC turnover data in Fig. 3a, the hazard function would not change in magnitude, but just in shape. Therefore, as far as the model calculations are concerned, the current removal by a random process would be subsumed into the removal by senescence, while the inferred extent of removal due to the parasite (directly and through the bystander effect) would remain the same. Hence, the blue and purple bars in Fig. 4 would be merged into one single senescence process which, however, would not change the main results and/or conclusions of this work which are predicated on the large difference between the extent of RBC removal by direct parasitization and by the bystander effect.

## Conclusions

A discrete recursive model of RBC dynamics in *M. mulatta* was developed that allowed, for the first time, the accurate quantification of the processes leading to the production and removal of RBCs in circulation under healthy conditions and during a *P. coatneyi* infection. The results support experimental evidence that removal of uninfected RBCs is the dominant process leading to malarial anaemia. The model suggests that the loss of uninfected RBCs is mostly due to an age-independent process, with only a small number of cells removed due to increased senescence. The mechanisms behind this high loss of uninfected RBCs during malaria are currently unknown and warrant further investigation.
